# Genotyping of suspected partial hydatidiform moles – our experiences and future directions

**DOI:** 10.3389/pore.2026.1612410

**Published:** 2026-05-25

**Authors:** Lajos Gergely, Vanda Repiska, Juraj Hutnik, Natalia Andova, Lubica Milosovicova, Miroslav Korbel, Liam McCullough, Alexandra Kristufkova, Ludovit Danihel, Helena Gbelcova, Petra Priscakova

**Affiliations:** 1 Institute of Medical Biology, Genetics and Clinical Genetics, Faculty of Medicine, Comenius University Bratislava, Bratislava, Slovakia; 2 Centre for Gestational Trophoblastic Disease of Slovak Republic, Bratislava, Slovakia; 3 1st Department of Gynaecology and Obstetrics, Faculty of Medicine, Comenius University Bratislava, Bratislava, Slovakia; 4 Institute of Pathological Anatomy, Faculty of Medicine, Comenius University Bratislava, Bratislava, Slovakia

**Keywords:** diploidy, DNA analysis, gestational trophoblastic disease, partial hydatidiform mole, triploidy

## Abstract

This brief research report summarizes our experience with the molecular differential diagnosis of clinically or pathologically suspected partial hydatidiform moles and discusses practical diagnostic options when economic resources or laboratory infrastructure are limited. We analyzed the genome composition of 68 suspected cases using short tandem repeat genotyping based on quantitative fluorescent PCR and fragmentation analysis. Genetic testing excluded a partial mole in 35 cases (51%) by demonstrating a diploid or monogynic monoandric diploid genome, while in 33 cases (49%) a partial mole was confirmed through identification of diandric triploidy (31 cases) or triandric tetraploidy (2 cases). No instances of digynic triploidy were detected in this cohort. Our findings indicate that, in the absence of access to a DNA laboratory, most suspected partial moles can still be accurately evaluated using ploidy assessment by more widely available methods, such as fluorescent *in situ* hybridization, DNA flow cytometry, or conventional karyotyping, given the apparent rarity of digynic triploidy among these cases.

## Introduction

The clinical and ultrasonographic picture, as well as the histopathological and immunohistochemical profile of partial hydatidiform moles (PHM) are often ambiguous, especially in the early weeks of pregnancy. For these reasons, the above-mentioned examinations alone are often not sufficient for the definitive diagnosis. The problems are the existence of numerous pathologies that can mimic partial moles. The histomorphological profile of the placenta of aneuploid pregnancies may include enlarged chorionic villi with surface invaginations, multifocal trophoblastic hyperplasia, stromal pseudoinclusions, and poorer vascularization, making it difficult to clearly distinguish them from partial moles [1–3].

Short Tandem Repeat (STR) genotyping is the most frequently utilized method for resolving the above-mentioned differential diagnostic dilemmas in developed countries, because it identifies the exact genome composition with the origin of different sets of chromosomes [4, 5].

Before the availability of STR genotyping, other genetic methods such as FISH (Fluorescent *In Situ* Hybridization), DNA flow cytometry, or classical karyotyping were used for these differential diagnostic purposes. These methods were replaced by STR genotyping because they assess only chromosomal ploidy and do not provide information on parental origin. Consequently, in cases of triploidy, digynic triploidy (which does not result in PHM) cannot be distinguished from diandric triploidy (the characteristic genomic constitution of PHM) [6–10].

STR genotyping is no doubt the most suitable method to distinguish partial moles from its mimics (like hydropic abortus, aneuploid conceptions…). However, it requires sophisticated laboratory infrastructure, which limits its accessibility in settings with restricted economic resources. This problem emphasizes the need to quantify the real prevalence of digynic triploidy among suspected PHM, so that the adequate, but also available method can be chosen depending on local infrastructure and options. The objective of this real-world study is to address this diagnostic challenge.

## Methods

From January 2020 to May 2025 there were 68 successfully genotyped cases in the Centre for Gestational Trophoblastic Disease of Slovak Republic. The study was approved by the Ethical Committee of University Hospital Bratislava - Saints Cyril and Methodius Hospital. The investigations were carried out following the rules of the Declaration of Helsinki of 1975, revised in 2013. We confirm that all published data have been anonymized in accordance with ethical standards.

The composition of the genome was determined using the Short Tandem Repeats (STR) genotyping technique, which involved Quantitative Fluorescent PCR (QF-PCR) and fragmentation analysis.

**FIGURE 1:**
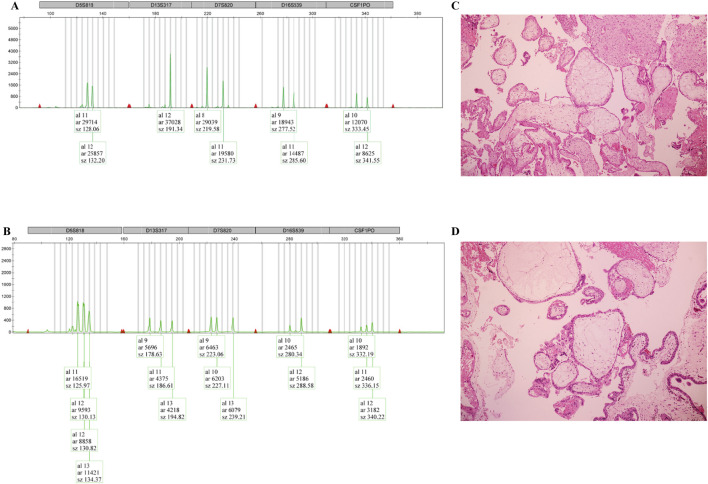
**(A,B)** STR fragmentation analysis of products of conception using the GenePrint® 10 System. Electropherograms show representative short tandem repeat (STR) profiles across multiple loci (including D5S818, D13S317, D7S820, D16S539, and CSF1PO). **(A)** Normal diploid product of conception (POC) showing a typical biparental inheritance pattern with one or two alleles per locus and balanced peak intensities consistent with diploid genome. **(B)** Partial hydatidiform mole demonstrating a diandric triploid pattern, characterized by the presence of three alleles at several loci and/or unbalanced peak height ratios consistent with two paternal and one maternal contribution. Allele calls (allele number, peak area, and fragment size in base pairs) are indicated below each peak. Red triangles denote internal size standard markers. **(C,D)** Histopathological comparison of missed abortion and partial hydatidiform mole (H&E staining). **(C)** Missed abortion showing focal hydropic degeneration of chorionic villi and absence of significant trophoblastic proliferation. **(D)** Partial hydatidiform mole characterized by a heterogeneous population of chorionic villi with focal hydropic degeneration and focal trophoblastic hyperplasia.

Depending on available material, manually microdissected chorionic villi and decidua from paraffin blocks, preparated-washed chorionic villi from curettage specimens and peripheral blood of the patient were used for DNA isolation with QIAGEN kits. QF-PCR was performed using the GenePrint 10 System (Promega) and Devyser Extend v2 (Devyser) commercial kits. Capillary electrophoresis was performed on ABI Prism 3130XL and ABI Prism 310 Genetic Analyzers. The recommended DNA concentration in reaction of samples isolated from formalin-fixed paraffin-embedded tissue were adjusted (increased) due to poor quality of DNA. The GeneMapper™ Software v4.1 was used for data analysis (Figure 1).

STR allelic profiles of the chorionic villi and the patient were compared. Monogynic monoandric diploid genome composition excluded partial mole, while diandric triploidy or triandric tetraploidy confirmed the diagnosis. In 9 cases, insufficient maternal DNA was available; therefore, the diagnosis of partial mole was excluded based solely on the diploid status of the chorionic villi.

### Statistical analysis

Descriptive statistics were used to summarize the number and proportion of confirmed and unconfirmed cases of partial hydatidiform mole across different referral sources. To assess whether the confirmation rate differed significantly by referral source (gynecologist-only, pathologist-only, both), a Chi-Square Test of Independence was applied. This test is appropriate for evaluating associations between categorical variables across multiple groups.

Given the relatively small sample size in the gynecologist-only group, pairwise post-hoc comparisons were also performed using Fisher’s Exact Test, which is more accurate for 2 × 2 contingency tables with small expected frequencies. This allowed us to identify specific group differences while maintaining statistical validity.

A p-value <0.05 was considered statistically significant. All analyses were performed using Microsoft Excel Python (SciPy library).

## Results

Of the 68 successfully genotyped cases, PHM were excluded in 35 cases (51%) based on a diploid or monogynic monoandric diploid genome, whereas it was confirmed in the remaining 33 cases (49%).

**FIGURE 2:**
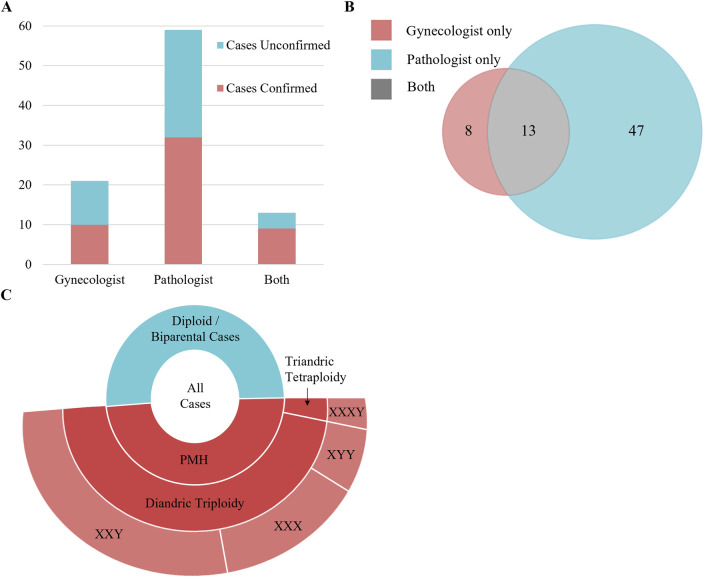
**(A)** Confirmation rate of PHM in referral groups. Confirmation rates varied significantly by referral type: 47.6% for gynecologist, 53.4% for pathologist, and 69.2% for combined referrals, with a statistically significant difference (χ^2^ = 6.39, df = 2, p = 0.0409). 13 cases that were suspected by gynaecologist and pathologist simultaneously are included in both referrer totals. **(B)** Referral source of 68 suspected PHM cases. **(C)** PMH was confirmed in 49% cases overall. Genetic composition of confirmed cases: 94% were diandric triploids (XXY: 58%, XXX: 29%, XYY: 13%) and 6% were triandric tetraploids (XXXY). No digynic triploidy was observed. PHM, partial hydatidiform mole.

In total, 21/68 (30.9%) cases were suspected by gynaecologists, 10/21 (47.6%) cases were confirmed. 60/68 (88.2%) cases were suspected by pathologists, 32/60 (53.4%) cases were confirmed. 13/68 (19.1%) cases from that cohort were suspected simultaneously by gynaecologist and pathologist and 9/13 (69.2%) were confirmed as PHM (Figure 2A).

To assess whether the confirmation rate differed significantly by referral source, a Chi-Square Test of Independence was performed. The test revealed a statistically significant difference in confirmation rates between referral groups (χ^2^ = 6.39, df = 2, *p* = 0.0409). This suggests that the likelihood of confirming a PHM diagnosis is associated with the type of referring specialist, with the higher confirmation rate observed in cases referred by both gynecologists and pathologists. To further explore these differences, pairwise post-hoc comparisons were conducted using Fisher’s Exact Test, which is more suitable for small sample sizes, referral categories were gynecologist-only (8 suspected cases, 1 case confirmed), pathologist-only (47 suspected cases, 23 cases confirmed) and group when both specialists suspected PMH (13 cases suspected, 9 cases confirmed) (Figure 2B).

The post-hoc analysis showed a statistically significant difference between the gynecologist-only and combined referral group (p = 0.0237), indicating that joint clinical and pathological suspicion increases diagnostic accuracy. However, the differences between gynecologist-only and pathologist-only (p = 0.1191), and between pathologist-only and both (p = 0.2257), were not statistically significant. These findings highlight the added value of multidisciplinary evaluation in improving the diagnostic yield for suspected PHM, particularly in ambiguous cases.

Of the 33 confirmed cases, 31 (94%) were diandric triploids, with sex chromosome complements XXY in 18 cases (58%), XXX in 9 cases (29%), and XYY in 4 cases (13%). Two cases (6%) were triandric tetraploids, both with an XXXY complement. Notably, all triploid cases were diandric, and no cases of digynic triploidy were observed in the cohort (Figure 1C).

Among the non-molar cases, two exhibited trisomy 16, two trisomy 13, one trisomy 21, and one a double trisomy involving chromosomes 16 and 22. These findings cannot be subjected to robust statistical interpretation, as aneuploidy testing was not performed systematically but rather on a case-by-case basis according to patient preference to determine the cause of pregnancy loss. This selection approach is inherently influenced by multiple confounding factors, including prior obstetric history and the patient’s emotional context.

## Discussion

Exact differential diagnosis, including genetic analyses is crucial for the appropriate management of patients with suspected partial hydatidiform moles. The fact that postmolar gestational trophoblastic neoplasia (invasive/metastatic hydatidiform mole or choriocarcinoma) can develop from PHM, justifies the need for dispensary care [11, 12]. 68 cases were analyzed based on the clinical suspicions of gynecologists, pathologists, or both of them. Genetic analysis excluded 51% of suspected PHM cases, indicating that reliance solely on gynecological and pathological evaluations would likely result in frequent overdiagnosis. Of the 33 confirmed partial hydatidiform moles, only 10 were clinically suspected by the gynecologist, indicating that approximately 70% of cases in our cohort were clinically silent. Given that histopathological findings are often subtle or inconclusive, the incorporation of genetic testing as a third diagnostic pillar is warranted to improve diagnostic confidence.

According to a relevant study by Nagy et al., none of histological parameters are unique to either diandric triploid gestation or digynic triploid gestation. According to their findings, in triploid cases with cistern formation combined with either trophoblastic hyperplasia or villous size ≥2.5 mm or syncytiotrophoblast lacunae a clear diagnosis of partial mole can be made, but in 35%–40% of diandric triploids this combination of histologic markers is not present [13].

Our analysis was limited by the inclusion of cases evaluated by multiple referring pathologists from independent institutions. As our department works with referral material, the diagnostic criteria used to distinguish non-molar hydropic abortions from partial hydatidiform moles were not fully uniform across cases. In addition, variability in the spectrum of assessed histopathological parameters, together with the inherently subjective nature of morphological interpretation, may have introduced inter-observer inconsistency.

Triploidy is a common lethal genetic pathology that affects approximately 1%–2% of all conception and in the majority of cases leads to first trimester miscarriage. The origin of the additional chromosome set can be paternal (diandric triploidy) or maternal (digynic triploidy). As a result of genomic imprinting, these two forms of triploidy display distinct biological characteristics, which are associated with varying maternal complications. Severe complications, like early second trimester gestational hypertension/preeclampsia, hyperemesis gravidarum, symptomatic hyperthyreosis and PHM with potential malignant transformation may occur in the diandric type [14]. All cases of triploidy in our patient group of suspected PHM were diandric - the digynic type was absent. We hypothesize that the absence of otherwise commonly observed digynic triploidy in our cohort reflects a differing—albeit partially overlapping—spectrum of clinical and histopathological features, resulting in a lower likelihood of referral of digynic triploid cases to a specialized trophoblastic disease center.

**FIGURE 3:**
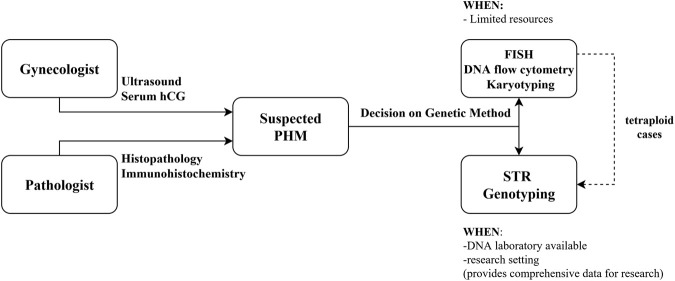
Diagnostic pathway for suspected PHM. Initial suspicion arises from gynaecological or pathological evaluation. Traditional methods (FISH, flow cytometry, karyotyping) are suitable when resources are limited. STR genotyping is preferred when available, and strongly recommended for rare tetraploid cases to distinguish triandric from biparental origin. hCG, human chorionic gonadotropin; PHM, partial hydatidiform mole; FISH, fluorescent *in situ* hybridization; STR, short tandem repeat.

In conclusion, these results indicate that in case of limited economical resources, where STR genotyping is not available, the diagnostic dilemma in the majority of histopathologically/clinically suspected PHM can be solved using older techniques detecting ploidy like FISH, DNA flow cytometry or classical karyotyping (Figure 3). Hence, decision to which method to use for genetic analyses of suspected PHM moles is primarily affected by available facility infrastructure, not specificity or sensitivity of the methods. If the facility already has cytogenetics infrastructure, there is no need for fast confirmation and probability of culture failure is low, classical karyotyping is an aeffective low-resource method. If fast confirmation of diagnosis is required, FISH and STR genotyping by QF-PCR are the best equivalent choices. However, in the cases of rare tetraploidy, STR genotyping is strongly advised to differentiate triandric from biparental tetraploidy.

## Data Availability

The original contributions presented in the study are included in the article/supplementary materials, further inquiries can be directed to the corresponding author.
